# Supplementary material for: Gene expression profile of sodium channel subunits in the anterior cingulate cortex during experimental paclitaxel-induced neuropathic pain in mice
Source: PeerJ. 2016 Nov 15;4:e2702. doi: 10.7717/peerj.2702 (PMC5119229; doi:10.7717/peerj.2702)
Supplement: Supplemental Information 1 [file peerj-04-2702-s001.docx]

| **Subunit** | **Animal number** | **1** | **2** | **3** | **4** | **5** | **6** | **7** | **8** | **9** | **10** | **11** | **12** |
| --- | --- | --- | --- | --- | --- | --- | --- | --- | --- | --- | --- | --- | --- |
| Nav1.1 | Control (Vehicle-treated) | 1.010374 | 0.6637087 | 1.491216 | 0.9931527 | 0.8722947 | 1.193698 | 0.9669998 | 0.8056639 | 1.241212 |  |  |  |
|  | Paclitaxel-treated | 2.125186 | 2.804070 | 1.658473 | 2.793013 | 3.083825 | 1.764962 | 1.310867 | 1.655581 | 1.720884 | 2.013474 | 2.344348 | 2.09084 |
| Nav1.2 | Control (Vehicle-treated) | 0.7105159 | 0.9237379 | 1.523623 | 0.3058597 | 1.554149 | 1.786260 | 1.177716 | 0.7665313 | 1.281219 | 1.018232 |  |  |
|  | Paclitaxel-treated | 1.171431 | 1.986698 | 10.528700 | 1.795442 | 2.790728 | 2.004499 | 2.550285 | 3.083121 | 12.198650 | 17.637250 | 9.972257 | 8.497961 |
| Nav1.3 | Control (Vehicle-treated) | 1.538154 | 0.6501299 | 1.089639 | 1.534356 | 1.131419 | 0.5286494 | 0.6076237 | 2.098633 | 0.9817879 | 0.7987504 |  |  |
|  | Paclitaxel-treated | 1.705250 | 1.940282 | 2.078727 | 2.588531 | 1.712911 | 1.107700 | 1.398422 | 4.978404 | 0.8008385 | 0.9109771 | 0.7105723 | 0.6593654 |
| Nav1.6 | Control (Vehicle-treated) | 0.201440 | 3.105460 | 1.598520 | 2.636410 | 1.199327 | 0.5831444 | 0.5423421 | 0.3349188 | 2.632956 | 0.8191408 | 1.384390 |  |
|  | Paclitaxel-treated | 3.273660 | 6.406510 | 7.348780 | 8.934200 | 2.875524 | 0.8540496 | 1.774433 | 5.252515 | 1.934709 | 2.154421 | 2.480991 | 2.655745 |

**Relative expression of mRNA for Na_v_1.1, Na_v_1.2, Na_v_1.3 and Na_v_1.6**
