# Supplementary material for: Gene expression profile of sodium channel subunits in the anterior cingulate cortex during experimental paclitaxel-induced neuropathic pain in mice
Source: PeerJ. 2016 Nov 15;4:e2702. doi: 10.7717/peerj.2702 (PMC5119229; doi:10.7717/peerj.2702)
Supplement: Supplemental Information 2 [file peerj-04-2702-s002.docx]

| **Subunit** | **Animal number** | **1** | **2** | **3** | **4** | **5** | **6** | **7** | **8** | **9** | **10** | **11** | **12** |
| --- | --- | --- | --- | --- | --- | --- | --- | --- | --- | --- | --- | --- | --- |
| Na_x_ | Control (Vehicle-treated) | 0.2296309 | 1.586600 | 2.744745 | 1.122021 | 0.6915474 | 3.014774 | 0.4274863 | 0.778474 | 0.6205506 | 0.694608 | 2.980155 |  |
|  | Paclitaxel-treated | 1.431842 | 9.368959 | 4.444555 | 7.969682 | 0.7930864 | 29.537990 | 6.796145 | 10.993410 | 1.443350 | 7.305861 | 4.828542 | 5.969544 |

**Relative expression of mRNA for Na_x_**
