# Supplementary material for: Gene expression profile of sodium channel subunits in the anterior cingulate cortex during experimental paclitaxel-induced neuropathic pain in mice
Source: PeerJ. 2016 Nov 15;4:e2702. doi: 10.7717/peerj.2702 (PMC5119229; doi:10.7717/peerj.2702)
Supplement: Supplemental Information 3 [file peerj-04-2702-s003.docx]

| **Subunit** | **Animal number** | **1** | **2** | **3** | **4** | **5** | **6** | **7** | **8** | **9** | **10** | **11** | **12** |
| --- | --- | --- | --- | --- | --- | --- | --- | --- | --- | --- | --- | --- | --- |
| Na_v_β1 | Control (Vehicle-treated) | 1.602334 | 1.128841 | 0.7142454 | 0.7740462 | 1.053331 | 1.577702 | 0.7274189 | 0.8272288 |  |  |  |  |
|  | Paclitaxel-treated | 1.504664 | 2.974076 | 3.926675 | 5.868161 | 0.9755301 | 2.136810 | 2.538292 | 2.405869 |  |  |  |  |
| Na_v_β2 | Control (Vehicle-treated) | 0.7314117 | 1.163460 | 1.175132 | 0.8417104 | 0.7117763 | 1.558973 | 1.070669 | 0.4620874 | 1.028022 | 0.6816899 | 3.088067 |  |
|  | Paclitaxel-treated | 1.793058 | 1.302987 | 2.021448 | 1.234239 | 1.342383 | 1.826687 | 3.525096 | 9.773639 | 0.2014003 | 0.6364581 | 0.8837259 | 0.5441479 |
| Na_v_β3 | Control (Vehicle-treated) | 0.8270926 | 1.904555 | 1.450638 | 1.076427 | 1.005491 | 0.6973235 | 1.324961 | 0.8850694 | 0.9034265 | 0.6368884 | 1.963661 |  |
|  | Paclitaxel-treated | 3.307110 | 6.401548 | 12.189810 | 10.230610 | 1.335472 | 2.861887 | 4.535830 | 6.874557 | 0.2368281 | 1.685191 | 1.952802 | 0.8793113 |
| Na_v_β4 | Control (Vehicle-treated) | 1.426333 | 0.807543 | 0.868187 | 0.7083843 | 1.726884 | 0.7271479 | 1.124204 | 1.424922 | 0.7670274 | 0.6004087 | 1.523881 |  |
|  | Paclitaxel-treated | 0.8744681 | 0.7534469 | 0.9295114 | 0.9475525 | 1.485712 | 1.385306 | 1.932771 | 6.458224 | 1.589842 | 1.362022 | 3.224443 | 3.191903 |

**Relative expression of mRNA for Na_v_β1, Na_v_β2, Na_v_β3 and Na_v_β4**
