# Supplementary material for: Gene expression profile of sodium channel subunits in the anterior cingulate cortex during experimental paclitaxel-induced neuropathic pain in mice
Source: PeerJ. 2016 Nov 15;4:e2702. doi: 10.7717/peerj.2702 (PMC5119229; doi:10.7717/peerj.2702)
Supplement: Supplemental Information 4 [file peerj-04-2702-s004.docx]

| **Subunit** | **Animal number** | **1** | **2** | **3** | **4** |
| --- | --- | --- | --- | --- | --- |
| Na_v_β1 | Untreated | 1.125409 | 0.9907548 | 0.9769794 | 0.9179898 |
|  | Vehicle-treated | 1.049317 | 0.9172583 | 0.8972923 | 0.9061991 |
| Na_v_β2 | Untreated | 1.090081 | 0.9084071 | 1.144303 | 0.8825098 |
|  | Vehicle-treated | 0.8671294 | 1.044354 | 0.9194507 | 0.981683 |
| Na_v_β3 | Untreated | 1.213238 | 0.9220472 | 0.9468531 | 0.9441006 |
|  | Vehicle-treated | 0.6970396 | 0.9554568 | 1.044717 | 0.6559067 |
| Na_v_β4 | Untreated | 1.077564 | 0.8352317 | 0.9524113 | 1.166609 |
|  | Vehicle-treated | 2.376036 | 1.845184 | 1.273308 | 1.644207 |

**Relative expression of mRNA for Na_v_β1, Na_v_β2, Na_v_β3 and Na_v_β4**
