# Supplementary material for: Gene expression profile of sodium channel subunits in the anterior cingulate cortex during experimental paclitaxel-induced neuropathic pain in mice
Source: PeerJ. 2016 Nov 15;4:e2702. doi: 10.7717/peerj.2702 (PMC5119229; doi:10.7717/peerj.2702)
Supplement: Supplemental Information 5 [file peerj-04-2702-s005.docx]

| **Subunit** | **Animal number** | **1** | **2** | **3** | **4** |
| --- | --- | --- | --- | --- | --- |
| Nav1.1 | Untreated | 1.381801 | 0.7215894 | 0.8860132 | 1.131942 |
|  | Vehicle-treated | 1.093611 | 0.9238625 | 0.9848323 | 0.9492763 |
| Nav1.2 | Untreated | 1.076874 | 0.7385154 | 1.335148 | 0.9417722 |
|  | Vehicle-treated | 0.7045383 | 0.7141258 | 0.7899592 | 0.936824 |
| Nav1.3 | Untreated | 0.988988 | 0.7158727 | 1.042412 | 1.354983 |
|  | Vehicle-treated | 1.187621 | 0.8537142 | 0.9153371 | 0.9379448 |
| Nav1.6 | Untreated | 1.324307 | 0.7437489 | 1.095119 | 0.927094 |
|  | Vehicle-treated | 1.324307 | 0.7437489 | 1.095119 | 0.927094 |

**Relative expression of mRNA for Na_v_1.1, Na_v_1.2, Na_v_1.3 and Na_v_1.6**
