# Supplementary material for: Gene expression profile of sodium channel subunits in the anterior cingulate cortex during experimental paclitaxel-induced neuropathic pain in mice
Source: PeerJ. 2016 Nov 15;4:e2702. doi: 10.7717/peerj.2702 (PMC5119229; doi:10.7717/peerj.2702)
Supplement: Supplemental Information 6 [file peerj-04-2702-s006.docx]

| **Subunit** | **Animal number** | **1** | **2** | **3** | **4** |
| --- | --- | --- | --- | --- | --- |
| Na_x_ | Untreated | 1.328329 | 0.8572052 | 1.484588 | 0.5915666 |
|  | Vehicle-treated | 0.6596682 | 0.8729752 | 0.7746478 | 0.5818675 |

**Relative expression of mRNA for Na_x_**
